# Do Oral Cholera Vaccine and Water, Sanitation, and Hygiene Combine to Provide Greater Protection Against Cholera? Results From a Cluster-Randomized Trial of Oral Cholera Vaccine in Kolkata, India

**DOI:** 10.1093/ofid/ofad701

**Published:** 2024-01-10

**Authors:** Justin Im, Md Taufiqul Islam, Faisal Ahmmed, Deok Ryun Kim, Birkneh Tilahun Tadesse, Sophie Kang, Farhana Khanam, Fahima Chowdhury, Tasnuva Ahmed, Md Golam Firoj, Asma Binte Aziz, Masuma Hoque, Hyon Jin Jeon, Suman Kanungo, Shanta Dutta, Khalequ Zaman, Ashraful Islam Khan, Florian Marks, Jerome H Kim, Firdausi Qadri, John D Clemens

**Affiliations:** International Vaccine Institute, Seoul, Republic of Korea; International Centre for Diarrheal Disease Research, Dhaka, Bangladesh; International Centre for Diarrheal Disease Research, Dhaka, Bangladesh; International Vaccine Institute, Seoul, Republic of Korea; International Vaccine Institute, Seoul, Republic of Korea; International Vaccine Institute, Seoul, Republic of Korea; International Centre for Diarrheal Disease Research, Dhaka, Bangladesh; International Centre for Diarrheal Disease Research, Dhaka, Bangladesh; International Centre for Diarrheal Disease Research, Dhaka, Bangladesh; International Centre for Diarrheal Disease Research, Dhaka, Bangladesh; International Vaccine Institute, Seoul, Republic of Korea; International Centre for Diarrheal Disease Research, Dhaka, Bangladesh; International Vaccine Institute, Seoul, Republic of Korea; Cambridge Institute of Therapeutic Immunology and Infectious Disease, School of Clinical Medicine, University of Cambridge, Cambridge, UK; ICMR–National Institute of Cholera and Enteric Diseases, Kolkata, India; ICMR–National Institute of Cholera and Enteric Diseases, Kolkata, India; International Centre for Diarrheal Disease Research, Dhaka, Bangladesh; International Centre for Diarrheal Disease Research, Dhaka, Bangladesh; International Vaccine Institute, Seoul, Republic of Korea; Cambridge Institute of Therapeutic Immunology and Infectious Disease, School of Clinical Medicine, University of Cambridge, Cambridge, UK; Madagascar Institute for Vaccine Research, University of Antananarivo, Antananarivo, Madagascar; Heidelberg Institute of Global Health, University of Heidelberg, Heidelberg, Germany; International Vaccine Institute, Seoul, Republic of Korea; International Centre for Diarrheal Disease Research, Dhaka, Bangladesh; International Vaccine Institute, Seoul, Republic of Korea; International Centre for Diarrheal Disease Research, Dhaka, Bangladesh; Fielding School of Public Health, University of California–Los Angeles, Los Angeles, California, USA; Vaccine Innovation Center, School of Medicine, Korea University, Seoul, Republic of Korea

**Keywords:** cholera, clinical trial, India, oral cholera vaccine (OCV), WASH

## Abstract

**Background:**

Oral cholera vaccine (OCV) and incremental improvements in household water, sanitation, and hygiene (WASH) within cholera-endemic areas can reduce cholera risk. However, we lack empiric evaluation of their combined impact.

**Methods:**

We evaluated a cluster-randomized, placebo-controlled trial of OCV (Shanchol) in Kolkata, India. The study population included 108 777 individuals, and 106 879 nonpregnant individuals >1 year of age were eligible to receive 2 doses of OCV or placebo. We measured cholera risk in all household members assigned to OCV vs placebo and in all members of households with “Better” vs “Not Better” WASH, where WASH was classified according to validated criteria. Protection was measured by Cox proportional hazard models.

**Results:**

Residence in an OCV household was associated with protective effectiveness (PE) of 54% (95% CI, 42%–64%; *P* < .001) and was similar regardless of Better (PE, 57%; 95% CI, 26%–75%; *P* = .002) or Not Better (PE, 53%; 95% CI, 40%–64%; *P* < .001) household WASH. Better WASH household residence was associated with PE of 30% (95% CI, 5%–48%; *P* = .023) and was similar in OCV (PE, 24%; 95% CI, −26% to 54%; *P* = .293) and placebo (PE, 29%; 95% CI, −3% to 51%; *P* = .069) households. When assessed conjointly, residence in OCV households with Better WASH was associated with the greatest PE against cholera at 69% (95% CI, 49%–81%; *P* < .001).

**Conclusions:**

These findings suggest that the combination of a vaccine policy and improved WASH reduces cholera risk more than either would alone, although the magnitude of either intervention was not affected by the other. Future randomized trials investigating OCV and WASH interventions separately and together are recommended to further understand the interaction between OCV and WASH.

## Teaser

In a cholera-endemic urban setting, the combination of improved household water, sanitation, and hygiene and a policy to vaccinate with oral cholera vaccine was associated with greater protective effectiveness against cholera in household residents when compared with either intervention alone.

## BACKGROUND

Cholera is a bacterial disease caused by ingestion of *Vibrio cholerae* harbored in contaminated water sources. Infection causes diarrhea and dehydration and can be fatal when left untreated. Cholera occurs as endemic and epidemic disease in resource-poor settings where clean water infrastructure and sanitation facilities are unavailable or compromised. Fortunately, there are effective and inexpensive tools to treat and prevent cholera. Improved methods of rehydration, including oral rehydration solution, have made deaths due to cholera rare, and the most recent generation of oral cholera vaccines (OCVs) has demonstrated strong and sustained protective effect against cholera. Despite these advances, cholera persists and continues to cause massive outbreaks globally. The Global Roadmap to End Cholera by 2030 [1] details the strategic use of OCV and improvements of water, sanitation, and hygiene (WASH) to help combat cholera. Major overhauls of WASH infrastructure at the municipal level, as experienced by wealthy urban centers during the 20th century, have reduced commonplace infectious diseases to marginal levels; however, evidence of the effectiveness of simple, inexpensive, and localized WASH interventions based on experimental clinical studies is sparse. Using innovative approaches, we have shown that naturally occurring variations in household WASH within cholera-endemic areas are associated with cholera risk in household residents [2, 3]. In principle, improved WASH reduces exposure to infectious inoculum in the environment and should enhance the ability of an effective vaccine to protection against disease or infection; in fact, evidence from a clinical study of OCV in Mirpur, Bangladesh, suggests that the impact of OCV may be enhanced by improvements in WASH [2]. As variations of naturally existing WASH differ from place to place and over time, we thought that it would be useful to evaluate the robustness of the findings from Bangladesh in a different setting. In this analysis, which takes advantage of a cluster-randomized controlled trial of OCV in Kolkata, India, we evaluate the combined impact of a policy to vaccinate in conjunction with attainable levels of improved household WASH in a cholera-endemic setting.

## METHODS

### Trial and Population

We evaluated a cluster-randomized, placebo-controlled trial of an inactivated OCV (Shanchol) in an urban slum in Kolkata, India, conducted between 2006 and 2011 [4–6]. The study population included 108 777 individuals who were present during a baseline census. The assessed population resided in 3933 dwellings, and each dwelling, which consisted of multiple households, was treated as a cluster. Demographic information and household WASH characteristics were collected at baseline. Individuals who later migrated into the study area and births occurring during the study period were excluded from analysis. Details of the trial have been published [4–6].

### Vaccination

Residents of the study area who were at least 1 year old and not pregnant at the point of assessment were eligible for dosing with OCV or placebo. Clusters were randomized to receive 2 doses of either OCV or heat-killed *Escherichia coli* K12 placebo. First and second doses were scheduled 14 days apart. The first round took place between 27 July and 13 August 2006 and the second round between 27 August and 10 September 2006. Randomized allocation of intervention was stratified by administrative ward (ward 29, 30, or 33) and dwelling size (≤20 or >20 residents). Allocation to OCV or placebo was concealed from participants and study staff.

### Diarrheal Surveillance

Passive surveillance for cholera was conducted in the study area for 5 years following vaccination. Residents from the study area presenting with diarrhea at 1 of the 9 participating clinics or 2 hospitals, representing all known sources of care for diarrhea, were diagnosed by stool culture. A diarrheal visit was defined as a visit where, in the past 24 hours, the patient reported ≥3 loose or liquid stools, at least 1 loose stool with blood, or 1 loose stool with evidence of dehydration. Multiple diarrheal visits, where ≤7 days separated the date of discharge from the date of subsequent symptom onset, were grouped into a single diarrheal episode. Rectal swabs were collected from patients with diarrhea and examined by culture methods at the National Institute of Cholera and Enteric Diseases for presence of *V cholerae*. Cholera was defined as a diarrheal episode where *V cholerae* 01 was isolated from at least 1 sample.

### Definition of WASH

As previously described, characteristics of household WASH were collected during a baseline census of the trial population [3]. These variables—dichotomized by having characteristics of “Better” or “Not Better” WASH—were included in a model that used recursive partitioning to design a decision tree that predicted the 5-year risk of cholera in members of households assigned to placebo in the trial. Assessed at baseline for the trial, a house with Better WASH had (1) access to safe sources of drinking water, (2) members reporting use of soap after defecation with a safe source of water for daily use, or (3) members reporting use of soap after defecation with use of private or shared flush toilet. A house with Not Better WASH had characteristics of (1) an unsafe source of drinking water and members who did not report handwashing with soap after defecation or (2) an unsafe source of drinking water, handwashing with soap after defecation, no access to private or shared flush toilet, and unsafe source of water for daily use.

### Analytic Approach

In this analysis, we assessed whether OCV and household WASH independently demonstrated a protective association against cholera and whether the magnitude of the protective effect of each intervention depended on the other intervention. We assessed all individuals residing in households assigned to OCV or placebo regardless of vaccination status to more closely estimate the public health impact in the population overall. Protection conferred by OCV was measured by comparing rates of culture-confirmed cholera diarrhea in residents of OCV households vs residents of placebo households, controlling for the WASH status of the household. Protection associated with household WASH was evaluated by comparing the relative rate of cholera in residents of households with Better WASH vs Not Better WASH, controlling for the vaccine assignment of the household. We then assessed if protection conferred by residence in an OCV household was modified by the status of WASH in the household. For this evaluation we analyzed OCV protection stratified by Better or Not Better household WASH status. Next, we assessed if the protective effect of household WASH was modified by assignment to either OCV or placebo household. For this evaluation we compared the effects of Better WASH separately by household assignment. Finally, we assessed cholera risk in each of 4 groups representing the different combinations of residence in OCV or placebo household and residence in Better or Not Better WASH household, using households assigned to placebo with Not Better WASH as the referent group.

### Statistical Analysis

We analyzed residents of households who were present at zero time, defined as the date of OCV or placebo second dose or the median date of second dose in the cluster of residence for nondosed individuals. Cholera cases were analyzed starting 14 days after zero time. Follow-up continued for up to 1825 days after zero time or until the first cholera event, migration out of the cluster, or death, whichever came first for the individual.

To evaluate protective effectiveness (PE), we fitted Cox proportional hazards regression models at the individual level, with the occurrence of cholera as the dependent variable, after verifying that the proportionality assumptions were fulfilled for independent variables. Models were adjusted for stratifying variables (ward of residence and cluster size), age, as well as covariates that were selected with a forward stepwise algorithm, based on a significance level of 5% for entry into the initial model and for being retained in the model. PE of OCV and Better WASH in the household was calculated as (1 – hazard ratio) × 100%, where the hazard ratio was estimated by exponentiation of the coefficient for the relevant variable in the model and the 95% CI was estimated with a robust sandwich method to account for household-level clustering. The threshold of significance for individual estimates of PE was *P* < .005 with a corresponding 95% CI (2-sided). The analysis was performed with the *survival* package for the Cox model, *car* package for collinearity, and *dplyr* package for data management in R Studio analytic software.

## RESULTS

### Assembly of the Trial Population

**Figure 1.**
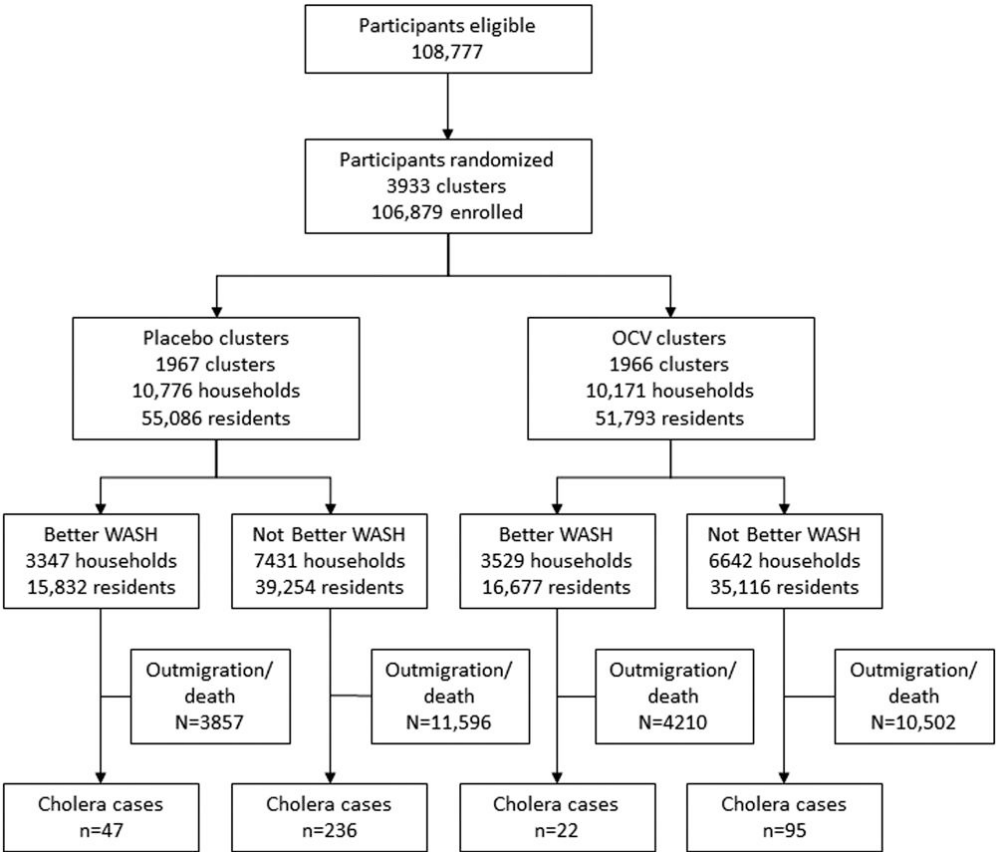
Flow diagram of study population randomized to oral cholera vaccine (OCV) or placebo. WASH, water, sanitation, and hygiene.

A total of 108 777 individuals were enumerated during the baseline census and 106 879 were enrolled in the study. Households were organized in 3933 dwellings, which were evaluated as clusters. Clusters were randomized 1:1 to receive either OCV or placebo: 1966 clusters with 10 171 households (n = 51 793 residents) were assigned to OCV, and 1967 clusters with 10 776 households (n = 55 086 residents) were assigned to placebo (Figure 1). Of the entire study population, 31 940 individuals received 2 doses of OCV and 34 969 received 2 doses of placebo. There were 400 cholera cases observed during 5 years of follow-up of the entire population enumerated at baseline. There were no substantive differences in baseline characteristics between the treatment arms (Table 1).

**Table 1.** Baseline Comparability of Members of OCV and Placebo Households

	Households, No. (%) or Mean ± SD			
	Overall (n = 106 879)	Placebo (n = 55 086)	OCV (n = 51 793)	*P* Value^a^
Better WASH	32 509 (30)	15 832 (29)	16 677 (32)	<.001
Age, y^b^				.500
0–4	6290 (6)	3248 (6)	3042 (6)	
5–14	19 569 (18)	10 156 (18)	9413 (18)	
≥15	81 020 (76)	41 682 (76)	39 338 (76)	
Age, y^b^	30 ± 18	30 ± 18	30 ± 18	.200
Gender				.093
Female	49 679 (46)	25 468 (46)	24 211 (47)	
Male	57 200 (54)	29 618 (54)	27 582 (53)	
Religion				
Others	40 144 (38)	20 081 (36)	20 063 (39)	<.001
Hindu	66 735 (62)	35 005 (64)	31 730 (61)	
Service holder have a stable job	47 948 (45)	24 280 (44)	23 668 (46)	<.001
Living in own house	36 221 (34)	18 673 (34)	17 548 (34)	>.9
Household expenditure^c^	3835 ± 4970	3771 ± 5706	3903 ± 4042	<.001
Longer than median distance to the clinic^d^	53 512 (50)	28 282 (51)	25 230 (49)	<.001

Abbreviations: OCV, oral cholera vaccine; WASH, water, sanitation, and hygiene.

^a^Pearson chi-square test; Wilcoxon rank sum test.

^b^Age in years measured at baseline.

^c^Expenditure calculated as average monthly expenditure in Indian rupees.

^d^Measured in meters as the minimum straight-line distance from household to closest study center.

### PE Against Cholera Associated With OCV Vaccination

To evaluate the PE associated with OCV vaccination, we compared the rates of cholera between placebo and OCV household residents. The incidence of cholera was 165 per 100 000 person-years (PY) in placebo households and 74 per 100 000 PY in OCV households. Residence in an OCV household was associated with a crude PE of 55% (95% CI, 43%–64%; *P* < .001) and an adjusted PE of 54% (95% CI, 42%–64%; *P* < .001; Table 2).

**Table 2.** Protective Effectiveness Against Cholera Associated With OCV Household Residence and Better WASH Over 5 Years

PE Associated With	No. of Participants (PY)	Cholera Episodes	Incidence^a^ (95% CI)	Crude PE (95% CI)	*P* Value	Adjusted PE (95% CI)	*P* Value
OCV residence							
Placebo	55 086 (172 033)	283	165 (145–184)	1 [Reference]		1 [Reference]	
OCV	51 793 (157 630)	117	74 (61–88)	55 (43–64)	<.001	54 (42–64)^b^	<.001
WASH							
Not better	74 370 (232 720)	331	142 (127–158)	1 [Reference]		1 [Reference]	
Better	32 509 (96 945)	69	71 (54–88)	50 (33–62)	<.001	30 (5–48)^c^	.023

Abbreviations: OCV, oral cholera vaccine; PE, protective effectiveness; PY, person-years; WASH, water, sanitation, and hygiene.

^a^Cases per 100 000 person-years.

^b^Adjusted by stratifying variables for randomization (ward of residence, cluster size), age group, service holder with a stable job, distance to the health center longer than median, and household WASH status.

^c^Adjusted by stratifying variables for randomization (ward of residence, cluster size), age group, service holder with a stable job, longer than median distance to the health center, and OCV allocation status.

### PE Against Cholera Associated With Household WASH

To evaluate the PE against cholera associated with residence in a Better WASH household, cholera incidence was compared between residents of Better and Not Better WASH households in the entire study population. The incidence of cholera was 142 per 100 000 PY in Not Better WASH households and 71 per 100 000 PY in Better WASH households. The corresponding crude PE associated with residence in a Better WASH household was 50% (95% CI, 33%–62%; *P* < .001) and the adjusted PE was 30% (95% CI, 5%–48%; *P* = .028; Table 2).

### PE Associated With Household WASH Stratified by Residence in an OCV Household

To evaluate whether the PE against cholera associated with household WASH varied by assignment to OCV or placebo, we evaluated OCV and placebo households separately. In placebo households, cholera incidence was 191 per 100 000 PY in Not Better WASH households and 97 per 100 000 PY in Better WASH households (crude PE, 49%; 95% CI, 27%–66%; *P* < .001; adjusted PE, 29%; 95% CI, −3% to 51%; *P* = .077). In OCV households, cholera incidence was 87 per 100 000 PY in residents of Not Better WASH households and 45 per 100 000 PY in Better WASH households (crude PE, 48%; 95% CI, 15%–68%; *P* = .009; adjusted PE, 24%; 95% CI, −26% to 54%; *P* = .293; Table 3).

**Table 3.** Protective Effectiveness Against Cholera Associated With Residence in a Better WASH Household Stratified by Assignment to Placebo or OCV

Vaccination: WASH	No. of Participants (PY)	Cholera Episodes	Incidence^a^ (95% CI)	Crude PE (95% CI)	*P* Value	Adjusted PE (95% CI)	*P* Value
Placebo							
Not Better	39 254 (123 803)	236	191 (166–215)	1 [Reference]		1 [Reference]	
Better	15 832 (48 230)	47	97 (70–125)	49 (27–66)	<.001	29 (−3 to 51)^b^	.069
OCV							
Not Better	35 116 (108 917)	95	87 (70–105)	1 [Reference]		1 [Reference]	
Better	16 677 (48 714)	22	45 (26–64)	48 (15–68)	.009	24 (−26 to 54)^c^	.293

Abbreviations: OCV, oral cholera vaccine; PE, protective effectiveness; PY, person-years; WASH, water, sanitation, and hygiene.

^a^Cases per 100 000 person-years.

^b^Adjusted by stratifying variables for randomization (ward of residence, cluster size), age group, household expenditure higher than median, and distance to the health center longer than median.

^c^Adjusted by stratifying variables for randomization (ward of residence, cluster size), age group, service holder with a stable job, household expenditure higher than median, and distance to the health center longer than median.

### PE Against Cholera Associated With Residence in an OCV Household Stratified by Household WASH Status

To evaluate if the PE against cholera associated with OCV household residence was affected by household WASH status, we evaluated residents of Better WASH and Not Better WASH households separately. In residents of Not Better WASH households, cholera incidence was 191 per 100 000 PY in placebo households and 87 per 100 000 PY in OCV households (crude PE, 54%; 95% CI, 41%–64%; *P* < .001; adjusted PE, 53%; 95% CI, 40%–64%; *P* < .001). In residents of Better WASH households, cholera incidence was 97 per 100 000 PY in placebo households and 45 per 100 000 PY in OCV households (crude PE, 54%; 95% CI, 20%–73%; *P* = .006; adjusted PE, 57%; 95% CI, 26%–75%; *P* = .002; Table 4).

**Table 4.** Protective Effectiveness Against Cholera Associated With OCV Vaccination Stratified by Residence in Better or Not Better WASH Household

WASH: Vaccination	No. of Participants (PY)	Cholera Episodes	Incidence^a^ (95% CI)	Crude PE (95% CI)	*P* Value	Adjusted PE (95% CI)	*P* Value
Not Better							
Placebo	39 254 (123 803)	236	191 (166–215)	1 [Reference]		1 [Reference]	
OCV	35 116 (108 917)	95	87 (70–105)	54 (41–64)	<.001	53 (40–64)^b^	<.001
Better							
Placebo	15 832 (48 230)	47	97 (70–125)	1 [Reference]		1 [Reference]	
OCV	16 677 (48 714)	22	45 (26–64)	54 (20–73)	.006	57 (26–75)^c^	.002

Abbreviations: OCV, oral cholera vaccine; PE, protective effectiveness; PY, person-years; WASH, water, sanitation, and hygiene.

^a^Cases per 100 000 person-years.

^b^Adjusted by stratifying variables for randomization (ward of residence, cluster size), age group, service holder with a stable job, and distance to the health center longer than median.

^c^Adjusted by stratifying variables for randomization (ward of residence, cluster size), age group, and distance to the health center longer than median.

### Combined Effects of OCV Assignment and Household WASH

We further evaluated the combined effects of OCV and household WASH by comparing the 4 possible categories of the 2 variables: (1) placebo household with Not Better WASH, (2) placebo household with Better WASH, (3) OCV household with Not Better WASH, and (4) OCV household with Better WASH. The incidence of cholera was highest in placebo households with Not Better WASH, 191 per 100 000 PY, and lowest in OCV households with Better WASH, 45 per 100 000 PY. Intermediate cholera incidence was observed in placebo households with Better WASH, 97 per 100 000 PY, and in OCV households with Not Better WASH, 87 per 100 000 PY (Table 5). When placebo households with Not Better WASH were set as the reference group, the adjusted PE was 27% (95% CI, −4% to 49%; *P* < .091) in residents of placebo households with Better WASH, 53% (95% CI, 40%–64%; *P* < .0001) in OCV households with Not Better WASH, and 69% (95% CI, 49%–81%; *P* < .001) in OCV households with Better WASH (Table 5).

**Table 5.** Protective Effectiveness Against Cholera by Residence in OCV or Placebo Household and by Residence in Better or Not Better WASH Household Considered Conjointly

Vaccination	WASH	No. of Participants (PY)	Cholera Episodes	Incidence^a^ (95% CI)	Crude PE (95% CI)	*P* Value	Adjusted PE^b^ (95% CI)	*P* Value
Placebo	Not Better	39 254 (123 803)	236	191 (166–215)	1 [Reference]		1 [Reference]	
	Better	15 832 (48 230)	47	97 (70–125)	49 (27–64)	<.001	27 (−4 to 49)	.091
OCV	Not Better	35 116 (108 917)	95	87 (70–105)	54 (41–64)	<.001	53 (40 to 64)	<.001
	Better	16 677 (48 714)	22	45 (26–64)	76 (62–85)	<.001	69 (49 to 81)	<.001

Abbreviations: OCV, oral cholera vaccine; PE, protective effectiveness; PY, person-years; WASH, water, sanitation, and hygiene.

^a^Cases per 100 000 person-years.

^b^Adjusted by stratifying variables (ward, cluster size), age group, service holder with a stable job, and distance to the health center longer than median.

## DISCUSSION

In this trial, cholera incidence was lower in households assigned to OCV as compared with placebo, and the relative reduction was similar regardless of whether the household of residence had Better WASH characteristics. Similarly, residents of Better WASH households had a lower incidence of cholera when compared with Not Better WASH households, and the reduction was similar in OCV and placebo households. We observed that the combination of OCV vaccination and Better WASH at the household level had the largest impact on cholera risk reduction, although the magnitude of the protective effect of each intervention did not depend on the other intervention.

Several important limitations should be considered when interpreting these findings. First, although our WASH variable was validated in the study population, it was constructed with simple household variables collected at baseline and may not adequately categorize households based on risk of cholera in household members. In addition, household characteristics were not updated during the study period; therefore, gradual upgrades to elements of WASH in these households were not accounted for. However, misclassification of household WASH would result in an underestimate of the true impact of WASH in the analysis. Second, WASH was not randomized in the population, and systematic differences in WASH may not be independent of cholera risk, thereby introducing bias into the results. We address this by controlling for potentially confounding variables in the analyses. Third, Better WASH may be idiosyncratic to the population under study and may not be generalizable to other settings. Fourth, our results included cases of cholera that produced clinical disease severe enough for patients to seek treatment. It is possible that there are important effects, such as the prevention of subclinical infections, that are not detectable by the study surveillance system. Finally, this analysis evaluates the impact of a vaccine in the entire household population. To better understand the independent and joint effects of OCV and WASH, it would be useful to separately assess recipients of OCV or placebo.

Our analyses have several important strengths. The data were analyzed from a prospective cluster-randomized trial of OCV in a well-defined population. Comprehensive follow-up for cholera diarrhea was conducted at 11 study centers and was led by a team of highly trained and experienced clinicians and microbiologists who made diagnoses without knowledge of vaccination or household WASH status. As well, we used a previously developed and validated definition of Better WASH household from the same study population [3].

Our findings are similar to a study from Dhaka, Bangladesh, where cholera risk reduction was greatest in OCV households with Better WASH, although protective effects were observed only when OCV recipients were assessed [7]. In both analyses, the magnitude of protection associated with OCV was not affected by household WASH status. This is despite the expectation that cleaner water sources and improved sanitation lower levels of circulating inoculum, leading to enhancement of vaccine protection. Few other studies have evaluated the independent and combined effects of OCV and WASH. The trial mentioned earlier included a combined program of OCV and WASH, but the analysis was limited by poor uptake of the WASH intervention [8]. A study conducted in Haiti demonstrated significant protection against cholera, although the study was not designed to evaluate the independent effects of OCV and WASH [9].

These findings suggest that attainable improvements in household WASH, which already exist within this poor urban slum setting, are associated with a reduction in cholera risk. Our study strengthens the notion that the combination of improved WASH and a policy to vaccinate reduces the risk of cholera more than WASH or vaccination alone; however, we did not show that the magnitude of protection offered by either intervention was affected by the other. We recommend further probing of this trial data set, including evaluating specific populations, such as only those who received 2 doses of OCV, and considering alternate clinical outcomes, such as severe cholera. To elucidate the interaction between OCV and WASH—the 2 main pillars of global cholera control—future randomized trials of OCV should consider evaluating a proven WASH intervention separately and with OCV.

## Notes

***Patient consent statement.*** The study protocol was approved by the ethics committee of the National Institute of Cholera and Enteric Diseases, the Health Ministry Screening Committee of India, and the International Vaccine Institute Institutional Review Board. Written informed consent was obtained from older residents and from the guardians of residents aged 1 to 17 years. Additional written assent was obtained from residents aged 12 to 17 years.

***Financial support.*** This work was supported by the Bill and Melinda Gates Foundation (INV-025386). The original trial was funded by the Bill and Melinda Gates Foundation through the Diseases of the Most Impoverished Program.
